# Mechanism of emulsion stabilized by an ultrasonically prepared protein–polyphenol-polysaccharide complex: structure, functional properties and interfacial behavior

**DOI:** 10.1016/j.ultsonch.2025.107622

**Published:** 2025-10-13

**Authors:** Yuyang Huang, Baoning Zheng, Bingyu Sun, Ying Zhu, Linlin Liu, Jiyuan Liu, Yixin Zhang, Yang Li, Xiuqing Zhu

**Affiliations:** a College of Food Engineering of Harbin University of Commerce, Key Laboratory of Food Science and Engineering of Heilongjiang Province, Key Laboratory of Grain Food and Comprehensive Processing of Grain Resource of Heilongjiang Province, Harbin 150076, China; b College of Food Science, Northeast Agricultural University, Harbin, Heilongjiang 150030, China

**Keywords:** Ultrasonic modification, Soybean isolate protein, (−)-Epigallocatechin gallate, Polydextrose, Emulsion, Interfacial adsorption

## Abstract

Based on the mechanism that polyphenols and polysaccharides can modulate protein conformation through non-covalent interactions such as hydrogen bonding and hydrophobic forces, the construction of ternary complexes offers a promising strategy for developing efficient emulsion-based delivery systems. This study employed soy protein isolate (SPI), (−)-epigallocatechin gallate (EGCG), and polydextrose (PD) as raw materials to fabricate SPI, SPI-EGCG, SPI-PD, and SPI-EGCG-PD complexes via ultrasonic treatment. These complexes were then used to stabilize emulsions, and their structural characteristics, functional properties, and interfacial behavior were systematically investigated. The results indicated that the protein secondary structure of the ultrasonicated samples underwent significant alterations compared to SPI and the complexes (SPI-EGCG, SPI-PD, SPI-EGCG-PD). Specifically, the contents of α-helix and β-sheet decreased significantly, while the content of random coils increased significantly. Also, their emulsifying capacity and antioxidant activity were both significantly enhanced (p < 0.05). The emulsion stabilized by the ultrasonically prepared SPI-EGCG-PD complex exhibited superior stability, as evidenced by the highest emulsion stability index (89.6 min), the smallest particle size (599.6 nm), the highest absolute ζ-potential value (−37.4 mV), the greatest interfacial adsorbed protein content (4.89 mg/mL), the best oxidative stability (5.31 μmol/L), the lowest interfacial tension (20.89 mN/m), and the best storage stability. This study elucidates the mechanism by which ultrasonic treatment promotes the synergistic adsorption of proteins, polyphenols, and polysaccharides. It further examines the role of the interfacial behavior of ultrasonicated proteins and their complexes in maintaining emulsion stability, thereby providing a theoretical foundation for developing highly stable delivery systems for bioactive substances.

## Graphical abstract

**Figure:**
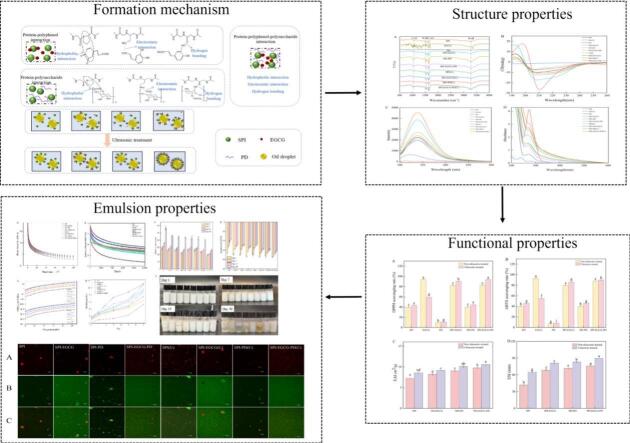

## 1 Introduction

Emulsions, due to the presence of grease, provide a key pathway to physically protect (reduce light and oxygen degradation) and improve the bioavailability of fat-soluble active ingredients, thus becoming an important delivery system for encapsulating fat-soluble active ingredients (e.g., carotenoids, curcuminoids, etc. [1,2]). To ensure that the emulsion has sufficient stability, must have a good emulsifier. Soy protein isolate (SPI), a widely available plant-based protein with amphiphilic properties, has been extensively used as a natural emulsifier. However, its large molecular size and relatively low surface hydrophobicity result in poor structural flexibility and weak interfacial activity, which limit its effectiveness in stabilizing emulsions [3]. In recent years, improving emulsion stability by designing protein–polyphenol/polysaccharide complexes has provided a solution for the preparation of long-term stable emulsions [4]. Compared with pure proteins, protein–polyphenol complexes increase the flexibility of protein molecules and improve the interfacial activity of proteins by providing additional hydrophobic sites, thus forming a thicker and more viscoelastic interfacial layer [5], but the emulsions prepared from protein-polyphenol complexes are less resistant to environmental factors (temperature, ionic strength, pH, etc.) [6]. In contrast, protein-polysaccharide stabilized emulsions are more stable [7], but their ability to prevent oxidative degradation of bioactives is limited. Therefore, ternary complexes based on proteins with surface activity, polysaccharides with spatial structure stability and polyphenols with antioxidant activity have gradually become the focus of research. For example, both ternary complexes, bovine serum albumin-dextran-gallic acid and pea isolate protein-high methoxyl pectin-EGCG, improved emulsion stability as emulsifiers compared to binary complexes [8]. EGCG possesses a tetracyclic structure with eight hydroxyl groups and is highly soluble in water, making it prone to form complexes with natural macromolecules [9]. Owing to its excellent antioxidant capacity and pharmacological properties, EGCG is commonly employed to modify proteins and enhance their functional attributes. PD, as a water-soluble dietary fiber, features a highly branched glucan backbone that can interact with the polar residues of SPI via hydrogen bonding. In addition, its β-1,6-glycosidic linkages endow the system with shear-thinning behavior [10]. PD not only provides steric hindrance to inhibit droplet aggregation but also avoids competition with polyphenols for protein binding sites.

Physical modification of proteins serves as an effective strategy for optimizing their functional properties to enhance emulsion stability. This approach allows for precise modulation of protein conformation and intermolecular interactions, thereby enabling the construction of efficient delivery systems. Currently, heat treatment, high-pressure homogenization, and ultrasonic are common techniques for physical modification of proteins. Among them, heat treatment is widely used, but it tends to trigger irreversible denaturation and aggregation of proteins, accelerating emulsion delamination and flocculation [11]. High-pressure homogenization improves solubility but decreases the emulsification of proteins [12]. In contrast, ultrasonic can disrupt the intermolecular forces of proteins, thereby altering the aggregation state of protein subunits and leading to changes in protein conformation [13], exposing the hydrophobicity of the molecules internally and reactive groups such as sulfhydryl groups, and enhancing the molecular flexibility of the proteins, thereby improving their emulsification properties [14]. Furthermore, the cavitation and mechanical shear forces generated during ultrasonication promote the partial unfolding of proteins and exposure of additional aromatic amino acids, thereby contributing to enhanced antioxidant capacity [15,16]. The complex formed between proteins and polyphenols exhibits potent antioxidant activity and improved surface activity, which helps anchor it at the oil–water interface where lipid oxidation occurs, thereby delaying lipid oxidation [17]. Furthermore, ultrasonic treatment promotes the exposure of hydrophobic regions within the protein, enhancing its binding capacity with polyphenols [18] and suppressing the generation of reactive oxygen species [19]. For example, ultrasonic reduced the α-helix content of proteins in SPI-EGCG complexes, increased molecular flexibility, and improved the antioxidant activity and emulsification properties of the complexes [20]. Compared to conventional heat treatment, Li et al [21] ultrasonically treated arachidonin-dextran/gum arabic complexes showed improved solubility and emulsification properties. However, there is little literature to date on the interfacial behavior and mechanism of enhancement of SPI-EGCG-PD ternary complexes by ultrasonic treatment.

Based on this, a ternary complex was prepared using ultrasound-assisted processing in this study. The effects of polyphenol and polysaccharide incorporation, as well as ultrasound treatment, on protein structure and functional properties were systematically evaluated through polyphenol binding capacity, Fourier-transform infrared (FTIR) spectroscopy, ultraviolet–visible (UV–Vis) absorption spectroscopy, fluorescence spectroscopy, circular dichroism (CD) spectroscopy, surface hydrophobicity, interfacial tension, emulsifying properties, and antioxidant activity. In addition, the emulsion systems were characterized in terms of particle size and ζ-potential, interfacial adsorbed protein content, rheological behavior, oxidative stability, and storage stability, in order to elucidate the role of interfacial behavior of the ternary complexes in maintaining emulsion stability. This work provides a theoretical foundation for developing novel stable food materials and promotes the application of ternary complexes in delivery systems.

## 2 Materials and methods

### 2.1 Materials

Soybean isolate protein (SPI) was self-extracted with a purity of 92.8 ± 0.31 %. (−)-epigallocatechin gallate(EGCG, ≥98 %), polydextrose(PD, ≥90 %), hydrochloric acid (HCl, 36 %), sodium hydroxide (NaOH, ≥99 %), sodium dodecyl sulfate (SDS, ≥98.5 %), 2,2-azidobis(3-ethylbenzothiazoline-6-sulfonicacid)(ABTS, ≥98 %), and 2,2-diphenyl-1-butyrylhydrazine (DPPH, ≥96 %), phosphate buffered saline(PBS). 8-aniline-1-naphthalenesulfonic acid (ANS), Kaomas brilliant blue (G-250), bovine serum albumin (BSA). Copper sulfate (CuSO4), sodium tartrate, soybean oil, Nile Red, Nile Blue were purchased from Sinopharm Chemical Reagent Co. Ltd (Beijing, China). All reagents were of analytical grade.

### 2.2 SPI extraction

According to the method of Yan et al [22], soybeans were ground and passed through a 60-mesh sieve, followed by defatting with hexane. The defatted flour was dispersed in deionized water (1:10, w/v), adjusted to pH 9.0 with 2 mol/L NaOH, and stirred for 1 h. After centrifugation at 9,000 rpm for 30 min, the supernatant was collected and adjusted to pH 4.5 with 2 mol/L HCl to precipitate proteins. The resulting precipitate was washed three times with deionized water (centrifugation at 6,500 rpm for 30 min per wash), redissolved by pH adjustment to 7.0 with 2 mol/L NaOH, and lyophilized using an ALPHA 1–2 LD plus freeze dryer (CHRIST, Germany). Protein content was determined to be 92.8 ± 0.31 % via the Kjeldahl method.

### 2.3 Sample preparation

#### 2.3.1 Preparation of SPI-EGCG complex

A 10 mg/mL SPI solution was hydrated overnight at 4 °C. EGCG was added to achieve a final concentration of 0.15 mg/mL, followed by pH adjustment to 7.0. The mixture was incubated for 2 h at 25 °C under light-protected and oxygen-free conditions. The resulting complex was dialyzed against deionized water at 4 °C for 48 h, then lyophilized.

#### 2.3.2 Preparation of the SPI-PD complex

Separate solutions of SPI (20 mg/mL) and PD (20 mg/mL) were prepared. Equal volumes were mixed, adjusted to pH 7.0, and incubated for 2 h at 25 °C under oxygen-free conditions. The resulting complex was dialyzed against deionized water at 4 °C for 48 h, then lyophilized.

#### 2.3.3 Preparation of the SPI-EGCG-PD complex

PD was incorporated into the pre-formed SPI-EGCG mixture to achieve a final PD concentration of 10 mg/mL. After pH adjustment to 7.0, the ternary mixture was incubated for 2 h at 25 °C under light-protected and oxygen-free conditions. The resulting complex was dialyzed against deionized water at 4 °C for 48 h, then lyophilized.

### 2.4 Ultrasonic treatment of samples

First, SPI, SPI-EGCG, SPI-PD, and SPI-EGCG-PD solutions were placed in an ice-water bath to ensure that the temperature of the protein solutions was below 20 °C during ultrasonic. Subsequently, the protein solutions were processed by a sonicator (Scientz Biotechnology Co., Ltd., Ningbo, China), and the titanium probe of the instrument was immersed in the solution 1 cm from the bottom of the beaker. The ultrasonic system worked for 5 s and rested for 5 s. The time was set to 10 min and the power was 400 W [23]. The reacted samples were dialyzed at 4 °C for 48 h and freeze-dried into powder for subsequent analysis. The samples were ultrasonic treated and labeled as SPI (U), SPI-EGCG (U), SPI-PD (U), and SPI-EGCG-PD (U). The non-ultrasonic-treated samples served as controls and were labeled as SPI, SPI-EGCG, SPI-PD, and SPI-EGCG-PD.

### 2.5 Structural characterization of complexes

#### 2.5.1 Determination of EGCG-binding equivalent

The EGCG content in the complexes was determined using the Folin–Ciocalteu method. Briefly, EGCG standard solutions were prepared at concentrations of 10, 20, 30, 40, and 50 mg/mL. Each standard solution was mixed with an equal volume of 1 mol/L Folin–Ciocalteu reagent (1:1, v/v), vortexed for 3 min, followed by the addition of 4 mL of 7.5 % (w/v) Na_2_CO_3_ solution to create an alkaline environment. The reaction was carried out in brown tubes for 2 h, and the absorbance was measured at 765 nm. Deionized water was used as the blank control. A standard calibration curve was constructed based on the absorbance values, with the resulting equation: Y = 0.0196x – 0.0265 (R^2^ = 0.9991). The lyophilized sample powders were dissolved to a final protein concentration of 1 mg/mL and treated in the same manner as the standard solutions. The polyphenol binding capacity was expressed as milligrams of EGCG per gram of conjugate (mg EGCG/g).

#### 2.5.2 Fourier-transform infrared spectroscopy

FT-IR spectra of the samples were recorded by using a Spectrum Two infrared spectrometer (PerkinElmer, Waltham, MA, USA). Before analysis, the lyophilized samples were pressed into 1–2 mm slices using a special mold. Then, FT-IR spectra were recorded in the range of 500–4000 cm^−1^ with a resolution of 4 cm^−1^.

#### 2.5.3 Circular dichroism spectroscopy

CD spectroscopy of all samples were recorded using a circular dichroism analyzer (Jasco-810, Jasco Corp., Tokyo, Japan). Spectroscopy were collected in the far-ultraviolet region of 190 ∼ 260 nm, and the protein concentration was adjusted to 0.5 mg/mL for the experiment. The secondary structure of the CD spectroscopy was calculated using online DichroWeb.

#### 2.5.4 Fluorescence spectroscopy

Fluorescence spectroscopy were measured using an F-6000 fluorometer (Hitachi Ltd., Tokyo, Japan), and the protein concentration was adjusted to 0.2 mg/mL. The measurement range was 300 ∼ 500 nm, the excitation wavelength was 280 nm, the scanning speed was 600 nm/min, the slit width was 5 nm, and the voltage was 600 V.

#### 2.5.5 Uv–visible absorption spectroscopy

UV–visible absorption spectroscopy were analyzed using a UV–visible spectrophotometer (UV-1700, Shimadzu Co., Ltd, Tokyo, Japan). The samples were dissolved in deionized water at a final concentration of 1 mg/mL. The UV–visible absorption spectroscopy were recorded in the wavelength range of 200 ∼ 600 nm.

#### 2.5.6 Surface hydrophobicity

The surface hydrophobicity of the samples was measured using an F-6000 fluorometer (Hitachi Ltd., Tokyo, Japan) using ANS as a fluorescent probe. The samples were diluted with phosphate buffer (10 mM, pH 7.0) to a protein concentration of 0.05–0.25 mg/mL. ANS solution (8 mM, 20 μL) was added to 4 mL of the samples, and the dark reaction was carried out for 15 min. The fluorescence intensity of the ANS probe was measured using a fluorometer with an emission wavelength of 200–600 nm and an excitation wavelength of 390 nm. The excitation and emission slit widths were 5 nm and 10 nm, respectively.

#### 2.5.7 Dynamic interfacial tension

The dynamic interfacial tension of the samples was measured using an OCA25 contact angle meter (OCA25, Dataphysics Instruments GmbH, Stuttgart, Germany) [24]. The samples were placed in a syringe with a sample volume of 16 μL. Soybean oil was added to optical glass test tubes, monitored with a video camera, and allowed to stand to achieve adsorption at the oil–water interface. The interfacial tension (γ) was calculated by droplet shape analysis according to the Young-Laplace equation. All measurements were performed at 25 °C.

(1) $$\pi \left(mN/m\right)=\gamma_{0}-\gamma$$

where γ_0_ denotes the distilled water–oil interfacial tension (48.92 mN/m) and γ denotes the complex-oil interfacial tension.

#### 2.5.8 Emulsification properties

The sample was dissolved in PBS(10 mmol/L, pH 7.0) to a final protein concentration of 1 mg/mL. The resulting solution was mixed with soybean oil at a 4:1 vol ratio (v/v) and homogenized. Subsequently, 50 μL of the emulsion was collected from the bottom layer and diluted with 5 mL of SDS solution (0.1 %, w/v). The absorbance of the diluted emulsion at 500 nm was measured at 0 min and 10 min, respectively. The emulsifying activity index (EAI) and emulsifying stability index (ESI) were calculated using the following equations:

(2) $$EAI\left(m^{2}/g\right)=\frac{2\times 2.303\times A_{0}\times DF}{10000\times \rho \times L\times \alpha }$$

(3) $$ESI\left(min\right)=\frac{A_{0}}{A_{0}-A_{10}}\times 10$$

where EAI is the emulsification activity, m^2^/g; ESI is the emulsification stability, min; 2 is the fixation factor; 2.303 is the reaction rate constant; A_0_ is the initial absorbance value; DF is the dilution factor; ρ is the mass concentration of the protein, g/mL; L is the light range, cm; α is the oil-phase occupancy ratio; and A_10_ is the absorbance value after 10 min of resting.

#### 2.5.9 Antioxidant activity

##### 2.5.9.1 DPPH radical scavenging rate

Anhydrous ethanol was used to dissolve 4 mg of DPPH powder, fixed to 100 mL, and configured into a fresh DPPH ethanol solution with a final concentration of 0.1 mmol/L. It was transferred to a brown volumetric flask and placed in a dark place for spare time (used within 3 h). DPPH was diluted with anhydrous ethanol to an absorbance value of 0.7 or less before sample analysis. Samples were dissolved in deionized water to a protein concentration of 1 mg/mL. A 2 mL aliquot was transferred to an amber centrifuge tube, mixed with an equal volume of freshly diluted DPPH solution, and vortexed well react for 30 min of dark incubation at 25 °C, absorbance was measured at 517 nm [1]. Zeroed with anhydrous ethanol, deionized water instead of sample solution was used as a blank control, and the DPPH radical scavenging rate was calculated as follows:

(4) $$DPPH\left(\%\right)=\frac{A_{0}}{\left(A_{0}-A_{t}\right)}\times 100\%$$

where A_0_ is the absorbance value of the sample at 517 nm and A_t_ is the absorbance value of the blank sample at 517 nm.

##### 2.5.9.2 ABTS free radical scavenging rate

The working solution of ABTS was prepared in advance by mixing 7 mmol/L ABTS solution and 2.45 mmol/L potassium persulfate solution in equal volume, stored at room temperature and protected from light for 12–16 h. The solution was diluted with deionized water to achieve an absorbance value of 0.70 ± 0.02 at 734 nm. Samples were dissolved in deionized water to a protein concentration of 0.5 mg/mL. A 1 mL aliquot of this solution was transferred into a light-protected centrifuge tube, and 3 mL of the diluted ABTS solution was added. The mixture was vortexed thoroughly and reacted at room temperature protected from light for 18 min. Absorbance was measured at 734 nm, with deionized water serving as blank control [25]. The formula for calculating the radical scavenging rate of ABTS is as follows.

(5) $$ABTS\left(\%\right)=\frac{A_{0}}{\left(A_{0}-A_{t}\right)}\times 100\%$$

where, A_0_ is the absorbance value of the sample at 734 nm and A_t_ is the absorbance value of the blank sample at 734 nm.

### 2.6 Preparation of emulsions

Under the condition of a protein concentration of 1 % (w/v), aqueous phases of SPI, SPI-EGCG, SPI-PD, SPI-EGCG-PD, as well as SPI (U), SPI-EGCG (U), SPI-PD (U), and SPI-EGCG-PD (U) complexes were prepared. After complete dissolution, the aqueous phase was mixed with the oil phase at a 9:1 vol ratio and homogenized using an IKA T25 homogenizer (IKA-Werke GmbH & Co. KG, Germany) at 12,000 rpm for 2 min to form a coarse emulsion. The coarse emulsion was then subjected to high-pressure homogenization at 60 MPa for 3 cycles to obtain the refined emulsion [22].

### 2.7 Emulsion characterization

#### 2.7.1 Particle size and ζ-potential

The emulsions were measured for particle size and and ζ-potential using a Zetasizer Nano laser particle size analyzer (Malvern Instruments, Malvern, UK). For particle size and zeta-potential measurements, the samples were diluted 400-fold and 250-fold, respectively, prior to analysis to minimize measurement errors caused by multiple light scattering. The analysis of each sample was repeated three times and the results were expressed as mean values with a refractive index of 1.460 for particles and 1.330 for dispersions.

#### 2.7.2 Interfacial adsorbed protein content

The method of Jiang et al [26] was used to determine the protein content in the emulsion. The emulsion sample was centrifuged at 10,000×*g* for 30 min, and the aqueous phase was collected using a syringe. This centrifugation and collection process was repeated three times to remove the emulsion layer. The aqueous phase obtained from the final cycle was collected, and the protein concentration was quantified using the Bradford method (Coomassie Brilliant Blue G-250) with bovine serum albumin (BSA) as the standard, by measuring the absorbance at 595 nm. The amount of protein adsorbed at the interface was calculated using the following formula:

(6) $$Interfacial\;adsorbed\;protein\;content\left(mg/ml\right)=C_{0}-C_{1}$$

where C_0_ refers to the concentration of total protein in the emulsion, mg/mL; C_1_ refers to the concentration of protein not adsorbed to the interface, mg/mL.

#### 2.7.3 Rheological properties

All rheological measurements were performed with an MCR102 Advanced Rotational Rheometer (Anton Paar Trading Co., Ltd., Graz, Austria), where the samples were loaded onto the rheometer measurement stage, allowed to equilibrate at 25 °C for 5 min, and viscosity measurements were performed at shear rates of 1–100 s^−1^. Vibration tests were performed in the linear viscoelastic region and the energy storage (G') and loss (G'') moduli relative to frequency were obtained using data analysis software [27].

#### 2.7.4 Laser scanning microscopy (CLSM)

The structural characteristics of the emulsions as well as the distribution of proteins and lipids were observed by confocal laser scanning microscopy (Leica Microsystems Inc., Heidelberg, Germany). Nile Blue solution (1 %, w/v) and Nile Red solution (0.1 %, w/v) were used to stain the aqueous and oil phases of the emulsions, respectively. For staining, 1 mL of emulsion was mixed with 25 μL of Nile Blue solution and 20 μL of Nile Red solution, and incubated for 30 min. The stained samples were then stored overnight at 4 °C. Observations were performed using a 60 × objective lens.

#### 2.7.5 Oxidative stability

The oxidative stability of the emulsions was evaluated by storing the samples at 37 °C in the dark for 12 days. Measurements were performed every 2 days.

##### 2.7.5.1 Thiobarbituric acid reactive substance assay (TBARS) assay

The TBARS content was used to characterize the secondary oxidation products of lipids. This method is based on the reaction between malondialdehyde (MDA), one of the primary oxidation products of lipids, and 2-thiobarbituric acid (TBA) to form a pink-colored complex. Briefly, 1 mL of emulsion sample was thoroughly mixed with 2 mL of trichloroacetic acid (TCA)-TBA-HCl reagent, which contained 0.25 mol/L HCl, 15 % (w/v) TCA, and 0.375 % (w/v) TBA. The mixture was heated in a boiling water bath for 15 min. After heating, it was cooled to room temperature for approximately 10 min. The cooled solution was then centrifuged at 9000 r/min for 10 min. After complete reaction, the absorbance of the supernatant was measured at 532 nm using a UV–visible spectrophotometer. The TBARS concentration was determined based on a standard curve prepared using 1,1,3,3-tetraethoxypropane [28].

#### 2.7.6 Storage stability

After preparing fresh emulsions, they were placed at room temperature to record the particle size and ζ-potential and appearance of the emulsion phase after regular storage periods (1, 7, 15, 30 days) [29].

### 2.8 Statical analysis

All experiments were repeated three times. Data are expressed as mean ± standard deviation. Data were statistically analyzed using one-way analysis of variance and significance was analyzed using Tukey’s test (p < 0.05). Origin 2019 software (OriginLab, Northampton, MA, USA) was used to plot data.

## 3 Results and discussion

### 3.1 Structural characterization of the complexes

#### 3.1.1 EGCG-binding equivalent

As shown in Fig. 2A, the polyphenol binding capacities of the SPI-EGCG, SPI-EGCG-PD, SPI-EGCG(U), and SPI-EGCG–PD(U) complexes were determined using the Folin–Ciocalteu method. The EGCG binding capacity of the SPI-EGCG complex was 9.94 mg EGCG·g^−1^ conjugate, while that of the SPI-EGCG-PD complex increased slightly to 10.57 mg EGCG·g^−1^ conjugate. This suggests that the presence of PD may promote further exposure of amino or thiol groups in SPI, thereby enhancing EGCG binding [30]. Notably, the ultrasonicated complexes exhibited significantly higher binding equivalent. The EGCG binding equivalent of SPI-EGCG(U) and SPI-EGCG-PD(U) reached 11.00 and 13.29 mg EGCG·g^−1^ conjugate, respectively. This enhancement is likely attributed to ultrasound-induced cavitation, which disrupts the β-sheet structure of SPI and exposes more EGCG binding sites [20]. Among all samples, SPI-EGCG-PD(U) exhibited the highest EGCG binding equivalent, which may directly contribute to its superior emulsion stability.

#### 3.1.2 Fourier-transform infrared spectroscopy

**Fig. 1:**
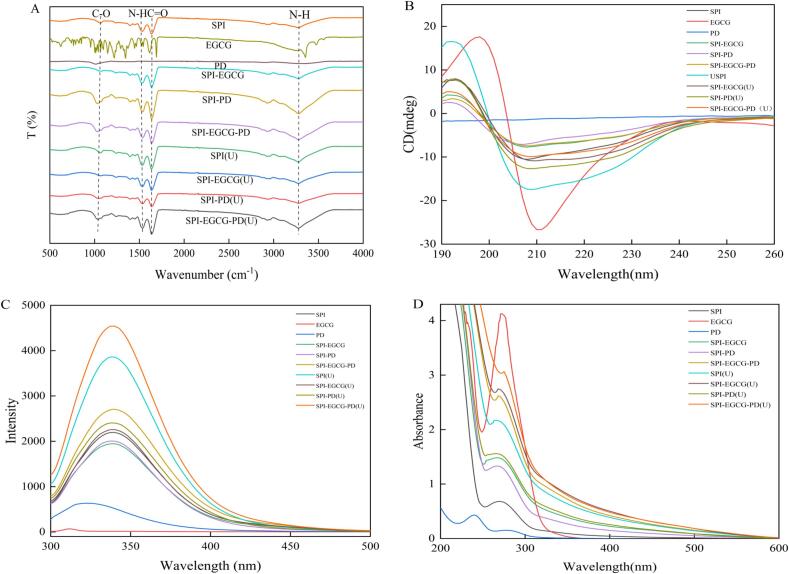
FT-IR spectroscopy (A), CD spectroscopy (B), Fuorescence spectroscopy (C), UV–visible absorption spectroscopy (D) of SPI, EGCG, PD, SPI-EGCG, SPI-PD, SPI-EGCG-PD and their ultrasonic-treated samples.

FT-IR can be used to assess changes in the chemical bonds formed between proteins, polyphenols, and polysaccharides. As shown in Fig. 1A, SPI showed characteristic absorption peaks in the amide A band (3273.09 cm^−1^, N-H stretching coupled with hydrogen bonding, O-H stretching, C-H stretching) and amide I band (1632.11 cm^−1^, C=O stretching). The characteristic peaks of EGCG at 1689 and 1613 cm^−1^ are attributed to C=O stretching vibrations, while the peaks at 3351 and 3471 cm^−1^ correspond to O-H stretching vibrations of phenolic hydroxyl groups. However, after binding with SPI, these typical EGCG peaks were no longer detected, indicating that molecular interactions had occurred between SPI and EGCG. Notably, the complex exhibited a significant blue shift at 3273 cm^−1^ with increased peak intensity, suggesting the formation of intermolecular hydrogen bonds. Similar results were reported by Zhang et al. [16], who observed a blue shift in the amide A band of lactoferrin-EGCG complexes due to enhanced hydrogen bonding between proteins and polyphenols. PD showed a broad absorption band near 1000 cm^−1^, attributed to the C–O–C stretching of its polysaccharide backbone. Compared with native SPI, the SPI-PD complex displayed stronger absorption in the range of 1030 ∼ 1100 cm^−1^. This phenomenon is attributed to structural alterations in SPI caused by the introduction of PD into the mixture, thereby resulting in the observed C–O stretching vibration [31]. Upon formation of the ternary complex, the internal molecular interactions of the protein were altered, leading to peak shifts in the spectrum. These spectral changes reflect the newly formed intermolecular interactions among protein, polyphenol, and polysaccharide, indicating that the combined action of EGCG and PD induces protein structural rearrangement and conformational transitions [32].

After ultrasound treatment, the amide I band of SPI(U) showed a red shift (1630 ∼ 1680 cm^−1^), suggesting changes in the position of peaks corresponding to protein secondary structure. Compared with the non-ultrasonic-treated binary complexes (SPI-EGCG and SPI-PD), both SPI–EGCG(U) and SPI-PD(U) exhibited blue shifts and enhanced intensity in the amide A band, implying that ultrasonic cavitation enhanced O-H and C-H vibrations, promoting hydrogen bonding between components [33]. Notably, the SPI-EGCG-PD(U) complex exhibited a blue shift to 1632 cm^−1^, indicating a transition in protein secondary structure from α-helix to β-sheet, which facilitates the aggregation of hydrophobic residues. This phenomenon is primarily attributed to protein reorganization induced by the combined effects of polyphenols, polysaccharides, and ultrasonic treatment [34]. Therefore, hydrophobic interactions and hydrogen bonds collectively induce the formation of the ternary complex.

#### 3.1.3 Circular dichroism spectroscopy

Circular dichroism is commonly used to probe changes in the secondary structure of proteins. As shown in Fig. 1B, compared to SPI, the SPI-EGCG complex exhibited slight changes in the intensity of the negative absorption band near 208 ∼ 220 nm. Whereas free EGCG displayed a strong CD signal in this spectral range, hence the spectral changes of SPI-EGCG can be attributed to induced non-covalent interactions and the conformational rearrangement of SPI driven by hydrogen bonding with EGCG [35]. In contrast, PD did not produce distinct characteristic spectral signals, indicating that the observed spectral changes in SPI-PD should be ascribed to the interaction between SPI and PD. Therefore, the multiple effects of EGCG, PD and ultrasonic altered the ellipticity of SPI, indicating significant changes in the secondary structure of the protein [20]. As shown in Table 1, complexation with EGCG decreased the α-helix content and increased the β-sheet content of SPI. This is because polyphenols were oxidized into quinones, which attacked the amino or sulfhydryl groups in SPI or the hydroxyl groups in phenolic compounds, thereby disrupting the stability of hydrogen bonds in SPI and leading to the unfolding of the protein's α-helix structure. This finding is consistent with the results reported by Feng et al. [36], who reported that the addition of EGCG led to a decrease in α-helix content and an increase in β-sheet content in ovalbumin. The addition of PD reduced the α-helix content and increased the β-sheet content in SPI, indicating a structural transition from α-helix to β-sheet and the formation of more compact complexes [37]. Compared to SPI and binary complexes, the SPI-EGCG-PD ternary complex exhibited a significant increase in random coil content. The elevated random coil structures may provide additional binding sites, thereby enhancing intermolecular interactions within the complex.

**Table 1:** Relative percentage content of secondary structures in the samples.

	α-helix (%)	β-sheet (%)	β-turn (%)	Random coil (%)
SPI	9.7 ± 0.06^d^	48.2 ± 0.06^c^	18.7 ± 0.14^c^	28.3 ± 0.10^e^
SPI-EGCG	8.4 ± 0.04^f^	49.1 ± 0.11^a^	18.1 ± 0.05^e^	28.9 ± 0.04^c^
SPI-PD	7.8 ± 0.05^h^	48.8 ± 0.05^b^	18.2 ± 0.04^e^	29.4 ± 0.09^a^
SPI-EGCG-PD	8.2 ± 0.08^g^	48.7 ± 0.05^b^	18.1 ± 0.06^e^	29.2 ± 0.04^b^
SPI (U)	14.2 ± 0.12^a^	42.0 ± 0.05^g^	19.6 ± 0.05^a^	28.6 ± 0.03^d^
SPI-EGCG (U)	10.1 ± 0.03^c^	46.9 ± 0.07^d^	18.5 ± 0.04^d^	29.0 ± 0.05^c^
SPI-PD (U)	10.8 ± 0.03^b^	44.6 ± 0.05^f^	19.1 ± 0.05^b^	29.4 ± 0.02^a^
SPI-EGCG-PD (U)	9.2 ± 0.12^e^	46.7 ± 0.07^e^	18.7 ± 0.05^cd^	29.6 ± 0.07^a^

Ultrasonic treatment significantly increased α-helix, β-turn, and random coil content while decreasing β-sheet structures, indicating disruption of the protein's internal architecture. This elevated random coil content likely correlates with exposure of hydrophobic amino acids such as tryptophan (Trp) and tyrosine (Tyr). Notably, in contrast to SPI (U) and binary complexes SPI-EGCG (U)/SPI-PD (U), the ternary complex SPI-EGCG-PD(U) demonstrated efficient α-helix to β-sheet conversion through synergistic EGCG-PD interactions, forming an ordered β-sheet-dominated network. This structural transition reflects ultrasound-induced transformation from compact conformations toward more flexible, interface-adsorption-favorable states, thereby enhancing composite stability through improved oil–water interfacial adsorption.

#### 3.1.4 Fluorescence spectroscopy

Intrinsic fluorescence reflects changes in protein tertiary structure by probing the microenvironmental polarity of hydrophobic amino acids, including Trp, Tyr, and phenylalanine (Phe). As shown in Fig. 1C, SPI exhibited a fluorescence λmax at 340 nm [38]. Neither free EGCG nor PD yielded significant fluorescence emission signals, indicating that the spectral changes observed in SPI-containing samples were not caused by the direct emission of EGCG or PD [39]. Consequently, the fluorescence intensity changes of the complexes can be attributed to microenvironmental alterations of SPI's intrinsic fluorophores (Trp or Tyr) following their interaction with EGCG or PD. SPI-EGCG exhibited reduced fluorescence intensity compared to SPI, suggesting potential masking of fluorophores through interactions between phenolic rings and Trp residues, leading to fluorescence quenching [40]. After binding with PD, the decrease in fluorescence intensity is attributed to the shielding effect of the polysaccharide chains [41]. The fluorescence intensity of the SPI-EGCG-PD ternary complex showed a certain degree of enhancement, accompanied by a slight red shift in the peak position. This may reflect a change in the polarity environment of Trp or Tyr residues from hydrophobic to hydrophilic. This phenomenon could be attributed to synergistic effects among multiple components causing protein conformational rearrangement [42].

The fluorescence intensities of all ultrasonicated samples were higher than those of their corresponding non-ultrasonicated groups, and the trend before and after ultrasonication was consistent, in the order: SPI-EGCG-PD(U) > SPI(U) > SPI-PD(U) > SPI-EGCG(U). This indicates that ultrasonication increased the number of exposed Trp groups within the protein [43], promoting more hydrophobic groups to be exposed to the exterior and enhancing hydrophobic interactions between components. This is consistent with the findings of Xue et al. [38], where ultrasonication exposed the hydrophobic regions of the SPI-cyanidin-3-galactoside complex.

#### 3.1.5 UV–visible absorption spectroscopy

Changes in UV–visible absorption spectroscopy can indicate interactions between proteins and small biomolecules. As shown in Fig. 1D, SPI exhibited characteristic absorption at 280 nm, attributed to Trp, Tyr, and Phe residues [44]. EGCG exhibited distinct UV absorption, whereas PD contributed only weak intrinsic absorption in this region. Compared to SPI, all complexes (SPI-EGCG, SPI-PD, SPI-EGCG-PD) showed significantly enhanced absorption intensity. This suggests that the increase is not merely due to the physical mixing of free EGCG and PD [45], but rather results from the formation of binary and ternary ground-state complexes between SPI and EGCG/PD [46]. Furthermore, SPI-EGCG displayed a shoulder peak at 254 nm, likely due to alterations in the microenvironment of Tyr and Trp residues caused by complexation with polyphenols/polysaccharides [47]. Cai et al. [48] found that interactions between amylase and procyanidins further altered the conformation of the protein molecule by exposing the aromatic heterocyclic hydrophobic groups within Tyr and Trp residues.

Ultrasonic disrupted SPI's β-sheet structures, exposing buried aromatic residues (Trp/Tyr), thus significantly increasing UV absorption intensity in SPI(U). Enhanced absorption in SPI-EGCG(U) and SPI-PD(U) complexes indicated ultrasonic-facilitated binding between proteins and polyphenols/polysaccharides. Upon formation of the ternary complex involving SPI, EGCG, and PD, ultrasonication further promoted the co-crosslinking of SPI by EGCG and PD to a greater extent, resulting in the highest UV absorption intensity for SPI-EGCG-PD(U) and the formation of a compact three-dimensional network structure.

#### 3.1.6 Surface hydrophobicity(H_0_)

**Fig. 2:**
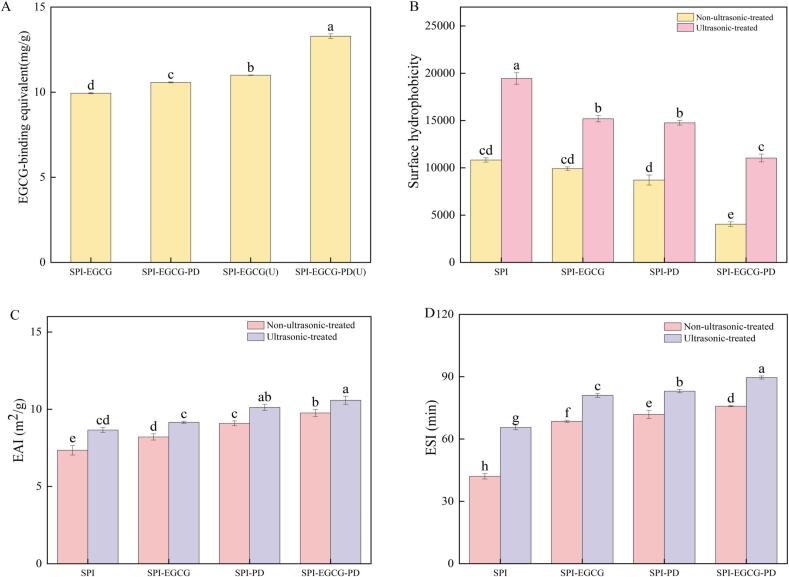
EGCG-binding equivalent(A) of SPI-EGCG, SPI-EGCG-PD and their ultrasonic-treated samples, surface hydrophobicity (B), emulsifying activity index(C), emulsifying stability index(D) of SPI, SPI-EGCG, SPI-PD, SPI-EGCG-PD and their ultrasonic-treated samples.

Surface hydrophobicity reflects the distribution of hydrophobic regions on protein surfaces, directly correlating with interfacial properties [49]. As shown in Fig. 2B, SPI-EGCG exhibited reduced surface hydrophobicity compared to SPI, indicating that cross-linked structures formed between polyphenols and proteins hinder partial unfolding, thereby limiting exposure of buried hydrophobic groups [50]. The SPI-PD complex showed significantly decreased hydrophobicity, likely due to the incorporation of highly hydrophilic PD molecules. This increases hydrophilic moieties on peptide chains, inducing chain extension and exposure of internal polar groups, consequently reducing hydrophobicity [51]. Compared to SPI-EGCG and SPI-PD complexes, SPI-EGCG-PD complexes show lower surface hydrophobicity. It may be due to the introduction of the polysaccharide PD and the polyphenol EGCG occupying the hydrophobic sites on the surface of the SPI, reducing the number of hydrophobic binding sites exposed on the protein surface, and impeding the binding of the ANS to proteins [41], indicating that the three of the SPI, with the EGCG and the PD, were successfully combined to form a ternary complex.

Ultrasonic treatment significantly alters the surface hydrophobicity of proteins and their complexes, a change directly linked to improved emulsifying properties. The cavitation and shear stress generated by ultrasonic cause the unfolding of SPI, exposing internal hydrophobic groups, and leading to a significant increase in its H_0_ [52]. Similarly, the SPI-EGCG and SPI-PD binary complexes also exhibited increased H_0_ after ultrasonic treatment. This is attributed to the unfolding of proteins or the dissociation of protein aggregates induced by ultrasonic, resulting in the exposure of previously buried hydrophobic groups. The increased H_0_ enhances the adsorption rate of proteins at the oil/water interface, which contributes to improved emulsifying properties [53]. After ultrasonic, the surface hydrophobicity of the SPI-EGCG-PD ternary complex reduces. This is attributed to the shielding by polyphenols and polysaccharide chains, combined with ultrasonic effects, which promote tight bonding among SPI, EGCG, and PD. They jointly prevent ANS from attaching to the complex's hydrophobic residues, thus lowering the H_0_ [54].

#### 3.1.7 Interfacial tension

To evaluate the stability of the samples, the interfacial tension (γ) was measured during the adsorption process, as shown in Fig. 4B. Within 10,000 s, γ gradually decreased. Emulsion stability is closely related to interfacial tension; a reduction in interfacial tension enhances emulsion stability [29]. When EGCG and PD were added to the SPI system, the interfacial tension decreased significantly. The SPI-EGCG-PD ternary complex exhibited the lowest γ value. This is likely because the three components engage in hydrogen bonding and electrostatic interactions, forming a relatively dense network structure that results in a more stable complex [29].

After ultrasonic treatment, the non-covalent bonds maintaining the native structure of SPI are disrupted. This leads to partial unfolding of its secondary structure and exposure of hydrophobic groups, enhancing the protein's surface hydrophobicity. Consequently, the protein adsorbs more readily to the oil–water interface, lowering the interfacial tension (γ) [55]. O'Sullivan similarly observed that the reduction in interfacial tension following ultrasonic treatment of SPI correlated with increased surface hydrophobicity [56]. The interfacial tension decreased for all complexes (SPI-EGCG, SPI-PD, SPI-EGCG-PD) after ultrasonic. This reduction occurs because ultrasonic treatment disrupts β-sheet structures, resulting in a looser protein conformation and exposure of hydrophobic groups. These exposed groups readily interact with hydrophobic interfaces, lowering γ. Simultaneously, enhanced structural flexibility accelerates molecular migration to the interface, ultimately manifesting as a decrease in interfacial tension [57]. The SPI-EGCG-PD ternary complex, after ultrasonic processing, exhibited the lowest interfacial tension and optimal emulsion stability.

#### 3.1.8 Emulsification properties

Emulsifying activity (EAI) and emulsifying stability (ESI) are commonly used to evaluate a protein's ability to form emulsions and stabilize fine oil droplets [58]. As shown in Fig. 2, Fig. 2, compared to the emulsifying activity (7.35 m2/g) and emulsifying stability (42.06 min) of SPI alone, the SPI-EGCG complex exhibits higher emulsifying activity (8.20 m2/g) and emulsifying stability (68.52 min). This improvement is likely due to the binding of EGCG's hydroxyl groups to amino acid residues on SPI during the reaction, which opens up the protein structure. This increased exposure enhances SPI's binding capacity with oil droplets, thereby improving emulsifying capability. Yan et al. [20] also reported similar results, indicating that binding EGCG to SPI can significantly improve its emulsifying properties. The higher emulsifying activity (9.08 m^2^/g) and emulsifying stability (71.83 min) of the SPI-PD complex compared to SPI may be due to the addition of higher molecular weight PD, which increases the solution viscosity of the mixed system and forms a stable network structure, thus enhancing emulsion stabilit [59]. The emulsifying activity (9.76 m2/g) and emulsifying stability (75.80 min) of the SPI-EGCG-PD ternary complex are significantly higher than those of SPI and its binary complexes. This suggests that interactions such as hydrogen bonding and hydrophobic forces between SPI, EGCG, and PD enhance the structural stability of the complex. This synergistic enhancement significantly improves the complex's adsorption capacity at the oil–water interface, thereby boosting emulsifying performance.

Compared to native SPI, the ultrasonicated SPI (U) showed improved ESI (65.60 min) and EAI (8.66 m^2^/g). This enhancement is primarily attributed to ultrasonic altering of the content of surface hydrophilic and hydrophobic groups within the protein, which facilitates the formation of emulsion droplets [60]. The EAI and ESI of both ultrasonicated SPI-EGCG (U) and SPI-PD (U) complexes were higher than their non-ultrasonicated complexes. This increase results from the cavitation effect of ultrasonic causing partial depolymerization of the proteins, exposing more functional groups (such as hydrophobic residues and active sites). This promotes rapid protein adsorption at the oil–water interface and enhances the spatial stability of the oil droplets [44]. When SPI, EGCG, and PD synergistically adsorb onto the oil–water interface through hydrophobic interactions and hydrogen bonding, the resulting interfacial film exhibits enhanced resistance to external shear forces and aggregation. Additionally, the cavitation effect of ultrasonic promotes the uniform dispersion of oil droplets and reduces aggregation and precipitation. These combined effects contribute to a significant improvement in emulsifying properties. Consequently, the ultrasonicated SPI-EGCG-PD (U) ternary complex exhibits the most outstanding emulsifying activity (10.58 m^2^/g) and emulsifying stability (89.6 min).

#### 3.1.9 Antioxidant activity

**Fig. 3:**
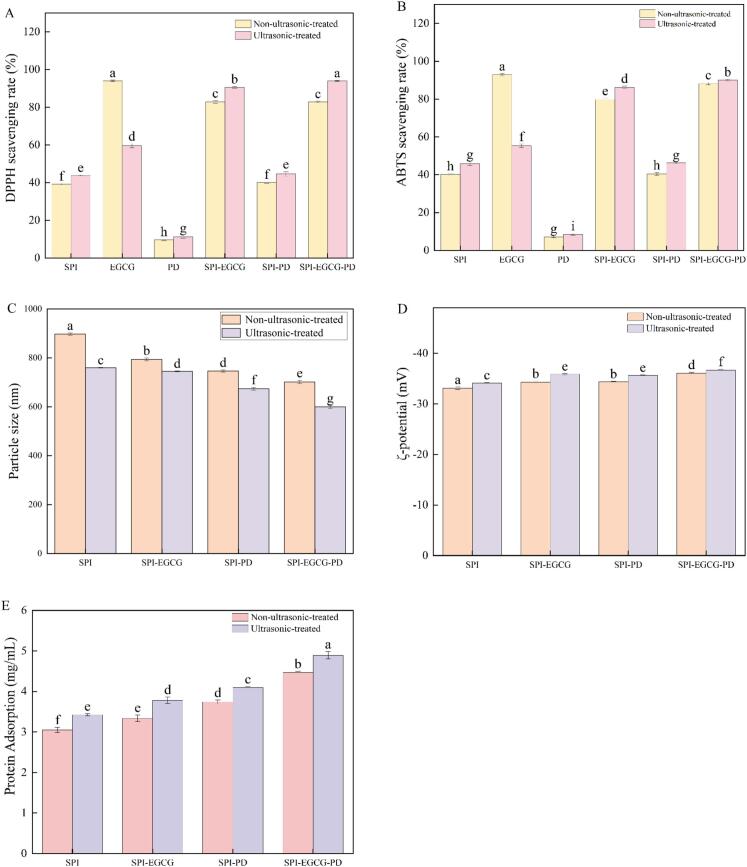
DPPH scavenging rate(A), ABTS scavenging rate (B) of SPI, EGCG, PD, SPI-EGCG, SPI-PD, SPI-EGCG-PD and their ultrasonic-treated samples and particle size(C), ζ-potential(D), interfacial adsorption protein content(E) of emulsions prepared using SPI, SPI-EGCG, SPI-PD, SPI-EGCG-PD, and their ultrasonic-treated samples.

The antioxidant properties of the prepared samples were evaluated by measuring their ability to scavenge DPPH and ABTS free radicals. As shown in Fig. 3, Fig. 3, EGCG contains multiple phenolic hydroxyl groups and exhibits high antioxidant activity, enabling it to scavenge free radicals [61]. EGCG demonstrates the highest radical scavenging activity, while SPI and PD exhibited weak scavenging capacity for both DPPH and ABTS radicals. After the addition of EGCG, the antioxidant capacity was significantly enhanced. This improvement is due to the introduction of more hydroxyl groups into SPI upon binding with EGCG. The addition of PD did not cause significant changes in the DPPH and ABTS scavenging rates of SPI. The antioxidant capacity of the SPI-EGCG-PD complex was significantly enhanced. This may be attributed to SPI acting as an emulsifier adsorbing at the interface, while EGCG and PD are anchored near the interface through interactions such as hydrogen bonding and hydrophobic forces, forming a dense antioxidant layer. The synergistic interaction among these three components enables the ternary complex to exhibit stronger antioxidant activity [62].

The significant increase in antioxidant activity of SPI and SPI-EGCG after ultrasonic treatment may be attributed to the mechanical waves and cavitation effects generated by ultrasonic. These effects can partially unfold the protein molecules, leading to changes in their secondary and tertiary structures. This unfolding exposes certain active groups originally hidden within the protein interior, thereby enhancing the protein's antioxidant activity. The antioxidant capacity of ultrasonicated PD showed a slight increase, while the antioxidant activity of ultrasonicated EGCG significantly decreased, likely due to ultrasonication damaging EGCG's functional groups. The ultrasonicated SPI-PD complex exhibited stronger free radical scavenging ability than its non-ultrasonicated complex. This suggests that ultrasonic may induce protein hydrolysis, generating bioactive peptides with higher antioxidant activity [63]. The significantly enhanced antioxidant capacity of SPI-EGCG-PD(U) is likely due to the improved binding ability of PD and EGCG to the protein after ultrasonic modification. This process incorporates more antioxidant functional groups into the system. This enhancement contributes to the further development of functional ingredient delivery systems with antioxidant properties for food applications.

### 3.2 Emulsion characterization

#### 3.2.1 Particle size and ζ-potential

As shown in Fig. 3C, the emulsion stabilized by SPI alone had the largest particle size (897.2 nm). In contrast, binary or ternary complexes formed between SPI and EGCG/PD improved the interfacial activity of SPI and lowered the interfacial tension. This enabled more effective distribution at the oil–water interface of the emulsion, overcoming droplet aggregation and forming a compact interfacial layer around the droplets [64]. Consequently, the droplets were dispersed more uniformly, resulting in a reduced particle size. The emulsions stabilized by ultrasonically prepared SPI and its complexes exhibited significantly smaller particle sizes than those stabilized by the non-ultrasonicated counterparts. This reduction is likely related to protein unfolding and the exposure of hydrophobic residues induced by ultrasonic cavitation, which promoted hydrophobic interactions between components [65]. The emulsion stabilized by SPI-EGCG-PD (U) exhibited the lowest particle size (599.6 nm). This can be attributed to ultrasonication promoting interactions (hydrogen bonding and hydrophobic interactions) between SPI, EGCG, and PD. These enhanced interactions led to the formation of complexes that stabilized emulsions with smaller droplet sizes. Smaller droplets possess a larger interfacial area, providing more sites for protein adsorption and thereby enhancing emulsion stability [66]. The reduction in particle size slowed down the droplet displacement rate and collision frequency, ultimately improving the stability of the ternary complex emulsion, as shown in Fig. 3D.

Generally, a higher absolute value of the ζ-potential indicates greater electrostatic repulsion between droplets, leading to enhanced emulsion stability. As shown in Fig. 3D, native SPI carries a negative surface charge (–33.1 mV). After complexation with EGCG, the absolute potential value increased significantly (−34.3 mV). This increase may be attributed to EGCG's inherent negative charge and the deprotonation of its phenolic hydroxyl groups under neutral conditions, contributing substantial negative charge. Silvio D. Rodríguez et al. [67]found that complexation of polyphenols with milk proteins can lower the protein's isoelectric point and increase the negative charge of the complex. The elevated absolute ζ-potential of SPI-PD (−34.3 mV) indicates enhanced electrostatic repulsion between particles, which can inhibit emulsion flocculation and sedimentation [23]. EGCG and PD occupy distinct binding sites on SPI, reducing steric hindrance and optimizing charge exposure. Consequently, the ζ-potential of the SPI-EGCG-PD complex increased significantly (−36.1 mV) [68]. Following ultrasonic treatment, the cavitation effect causes protein structures to unfold, exposing hydrophobic residues and charged groups [69]. Therefore, the emulsion prepared with ultrasonicated samples exhibited a significantly higher absolute zeta potential value compared to that prepared with non-ultrasonicated samples. Generally, a larger absolute ζ-potential value indicates greater electrostatic repulsion between droplets, leading to greater emulsion stability. Therefore, the ζ-potential results demonstrate that the emulsion stabilized by SPI-EGCG-PD (U) is more stable.

#### 3.2.2 Interfacial adsorbed protein content

Interfacial adsorbed proteins influence the flocculation and coalescence of emulsions. Generally, an increase in the amount of interfacial adsorbed protein enhances the protein's ability to adsorb at the oil–water interface, thereby stabilizing the emulsion [70]. As shown in Fig. 3E, compared to SPI (3.05 mg/mL), the interfacial adsorbed protein content of SPI-EGCG (3.34 mg/mL) was significantly higher. This may be attributed to the phenolic hydroxyl groups of EGCG binding with the polar groups of SPI, forming a dynamic cross-linking network. This promotes the adsorption and rearrangement of the protein at the oil–water interface, enhancing its interfacial adsorption capacity and further optimizing interfacial adsorption behavior [57]. PD likely increases the interfacial adsorbed protein content of SPI-PD (3.74 mg/mL) by inhibiting the formation of insoluble aggregates. The SPI-EGCG-PD ternary complex exhibits synergistic adsorption, resulting in an increased amount of interfacial adsorbed protein (4.46 mg/mL), thereby collectively stabilizing the emulsion.

Ultrasonic treatment moderately unfolds the structure of SPI, exposing internal hydrophobic groups. This enhances the surface hydrophobicity of the protein, thereby strengthening its migration rate and adsorption propensity at the oil–water interface. This structural change facilitates protein adsorption at the oil–water interface, increasing the interfacial adsorbed protein content (3.43 mg/mL). Ultrasonic also reduces the particle size and yields a more uniform distribution in the binary and ternary complexes, minimizing intermolecular aggregation. Consequently, a denser adsorption layer forms at the oil–water interface. Furthermore, the cavitation effect of ultrasonic promotes non-covalent interactions between SPI, EGCG, and PD, while potentially inducing the formation of some covalent bonds (such as C-S and C-N bonds). These effects collectively lead to a further increase in the interfacial adsorbed protein content (4.89 mg/mL), thereby enhancing the interfacial stability of the complexes [71].

#### 3.2.3 Rheological properties

**Fig. 4:**
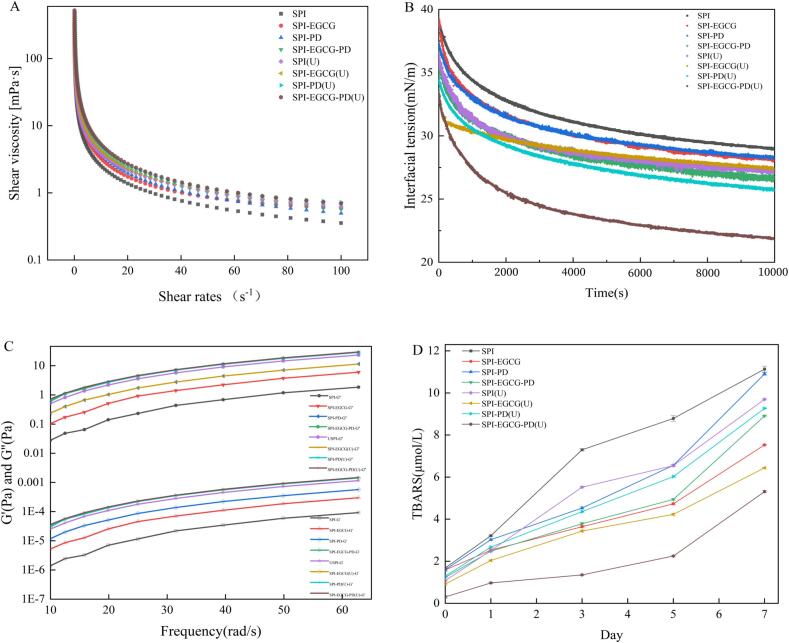
Shear viscosity(A), interfacial tension (B),storage modulus and loss modulus(C), oxidative stability(D) of emulsions prepared using SPI, SPI-EGCG, SPI-PD, SPI-EGCG-PD, and their ultrasonic-treated samples.

Apparent viscosity is a crucial indicator for assessing the stability of emulsion systems. As shown in Fig. 4A, within the shear rate range of 0.1 ∼ 100 s^−1^, the viscosity of the emulsions gradually decreased and eventually stabilized. This indicates that all samples exhibited distinct shear-thinning behavior. The SPI-EGCG and SPI-PD complexes enabled the formation of a thicker adsorbed layer at the oil–water interface, which increased the viscoelasticity of the oil droplet surface and resulted in higher macroscopic viscosity of the stabilized emulsions. Compared to SPI and the binary complexes, the emulsion stabilized by SPI-EGCG-PD showed increased apparent viscosity. This enhancement is likely due to the interactions among the protein, polyphenol, and polysaccharide, enabling the formation of a tighter three-dimensional protein network film at the emulsion interface [72]. The emulsions prepared with ultrasonicated complexes showed an increase in apparent viscosity. This is because ultrasonication typically reduces the particle size of the complexes, leading to a more uniform and denser distribution of complexes at the interface. This increases the emulsion's resistance to flow, resulting in higher viscosity for emulsions stabilized by ultrasonicated complexes. As the apparent viscosity increases, the motion rate of the emulsion slows down. This effectively suppresses aggregation and flocculation caused by droplet collisions, thereby enhancing the stability of the emulsion [73].

Measuring the storage modulus (G') and loss modulus (G“) is commonly used to characterize the viscoelastic behavior of emulsions, providing simultaneous information about their fluid-like or solid-like characteristics. As shown in Fig. 4C, the G” of all emulsions was significantly higher than G', indicating the formation of a flow-like liquid structure. Compared to SPI, emulsions stabilized by SPI-EGCG exhibited a significant increase in both the storage modulus (G') and loss modulus (G''). This enhancement is attributed to polyphenols strengthening the mechanical strength of the interfacial film through hydrogen bonding and hydrophobic interactions, leading to the formation of a denser interfacial film at the oil–water interface. PD forms a viscoelastic network within the continuous phase, inhibiting droplet aggregation through steric hindrance and increasing the viscosity of the continuous phase [74]. Furthermore, the β-1,6-glycosidic bonds of PD facilitate its co-adsorption with SPI at the interface, contributing to a thicker interfacial layer, thereby further increasing the emulsion's G' and G''. When EGCG and PD occupy distinct binding sites on SPI, they form a denser and more stable three-dimensional network structure [72]. This synergistic interaction between the two components results in a significant increase in G' and G'' for emulsions stabilized by SPI-EGCG-PD. The improvement in both G′ and G″ of the emulsion prepared with the ultrasonicated sample can be attributed to the cavitation and shear effects of ultrasound, which significantly reduce the particle size of the complexes. This leads to a more uniform droplet distribution, thereby promoting the adsorption of a greater number of complex molecules at the interface and facilitating the formation of a denser and more continuous interfacial film [72]. The SPI-EGCG-PD (U) complex-stabilized emulsion showed a larger increase in G', indicating a shift in the system's behavior from viscous to elastic dominance. In conclusion, the SPI-EGCG-PD (U) complex, through multicomponent synergy and ultrasonication, significantly enhances the rheological properties of the ternary complex-stabilized emulsion. This promotes the formation of a compact and stable interfacial layer, granting the emulsion distinct advantages in stability.

#### 3.2.4 Confocal laser scanning microscopy (CLSM)

**Fig. 5:**
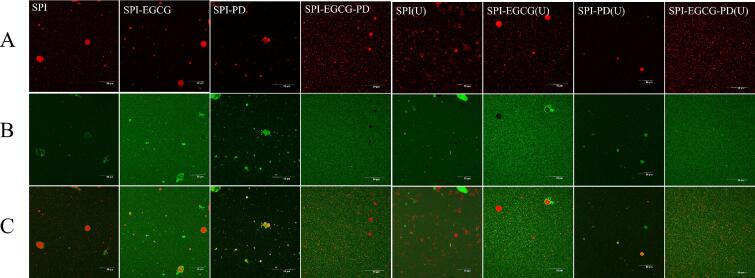
Laser confocal microscopy image of emulsions prepared using SPI, SPI-EGCG, SPI-PD, SPI-EGCG-PD, and their ultrasonic-treated samples, oil distribution (A), protein distribution (B), overlapping image (C).

The interfacial structure of the emulsions was observed using confocal laser scanning microscopy (CLSM) by labeling the complex particles and oil phase. As shown in Fig. 5, the SPI-stabilized emulsion exhibited large, irregularly shaped oil droplets with uneven protein coverage, indicating poor interfacial adsorption and weak emulsifying capacity. With the addition of EGCG, the oil droplet size decreased, and protein distribution became more uniform. Emulsions stabilized by the SPI-PD complex showed further improved dispersion, inhibiting droplet aggregation through steric hindrance and thereby stabilizing the emulsion [75]. Notably, the SPI-EGCG-PD ternary system in non-ultrasonicated samples displayed the most uniform microstructure: red oil droplets surrounded by green protein solution without significant coalescence, indicating the formation of a compact and integrated interfacial network.

Ultrasonic treatment induces rearrangement and reorganization of the oil droplets surrounding the complexes, resulting in a more uniform distribution within the emulsion and thereby enhancing its stability. Significantly, ultrasonicated SPI-EGCG-PD complexes generated smaller droplets with a larger interfacial area, providing more sites for protein adsorption. The interaction between oil droplets and complexes was strengthened, thereby improving emulsion stability and viscoelasticity [66]. Adsorption of SPI-EGCG-PD (U) at the oil–water interface formed a densely packed layer, acting as a physical barrier to enhance emulsion stability.

#### 3.2.5 Oxidative stability

Generally, the longer the storage time of an emulsion, the more severe the lipid oxidation becomes. Malondialdehyde (MDA), a representative secondary oxidation product of lipids, reflects the rate and intensity of lipid peroxidation. As shown in Fig. 4D, the TBARS values of all emulsion groups increased with storage time, indicating that lipids in the emulsions underwent oxidation during storage, generating secondary oxidation products [74]. The SPI-stabilized emulsion exhibited the highest TBARS values, demonstrating its insufficient protective capability at the oil–water interface, making it prone to oxidative degradation. In contrast, the significantly lower lipid oxidation levels observed in SPI-EGCG and SPI-EGCG-PD emulsions were attributed to the introduction of EGCG. EGCG provides a phenolic hydroxyl antioxidant barrier, effectively inhibiting free radical generation [16]. The TBARS values of the SPI-PD emulsion were lower than those of SPI but still higher than those of EGCG-containing complexes. This reduction might be related to the spatial structural effects of PD. However, due to PD's lack of free radical scavenging capacity, its overall antioxidant effect remained weaker than that of EGCG-containing samples.

The TBARS values of all ultrasonicated samples were lower than their corresponding non-ultrasonicated groups. This trend can be attributed to ultrasonication promoting the tight synergistic adsorption of protein, polyphenol, and polysaccharide at the oil–water interface. This forms a compact and homogeneous interfacial film, reduces oil droplet aggregation, and enhances physical barrier properties [63]. Furthermore, the emulsion prepared with the ultrasonicated complex exhibited a more homogeneous distribution, thus effectively reducing droplet–droplet collisions and coalescence, and consequently suppressing lipid oxidation. Notably, the SPI-EGCG-PD (U) emulsion exhibited the lowest TBARS values after 7 days. This can be attributed to the synergistic effect between the compact interfacial structure and the EGCG-rich antioxidant layer, thus providing more effective protection against oxidative degradation. The experiment demonstrates that the SPI-EGCG-PD ternary complex, under ultrasonic modification, possesses superior oxidative stability.

### 3.3 Storage stability

**Fig. 6:**
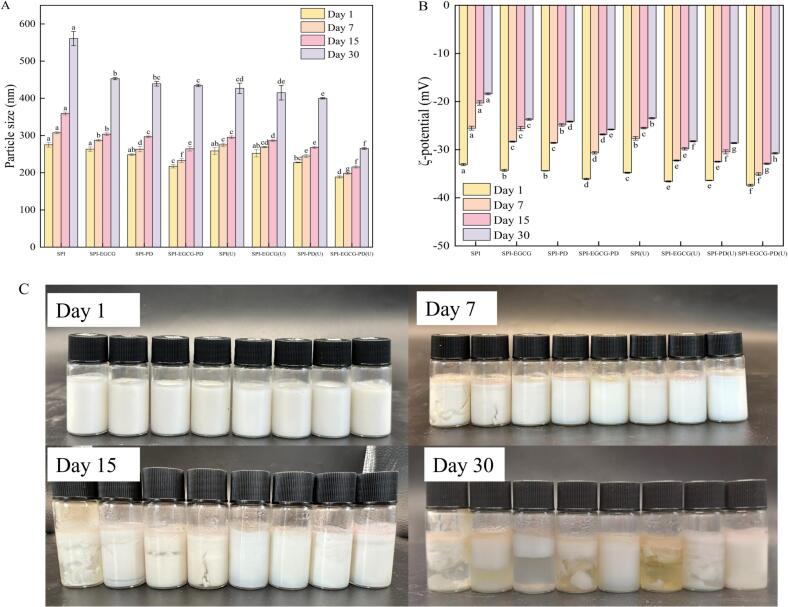
Particle size(A), ζ- potential(B), appearance(C) changes during storage of emulsions prepared using SPI, SPI-EGCG, SPI-PD, SPI-EGCG-PD, and their ultrasonic-treated samples.

The change in particle size during storage is a well-established indicator for evaluating the physical stability of emulsions. As shown in the Fig. 6, Fig. 6, the particle size of all samples increased significantly with prolonged storage time. Notably, the emulsion stabilized by SPI-EGCG-PD(U) exhibited the smallest increase in size and maintained the lowest particle size (nm) after 30 days, demonstrating superior storage stability. This enhanced stability can be attributed to the ultrasound treatment, which effectively induces partial unfolding of the SPI structure, increases molecular flexibility, and exposes additional hydrophobic regions. These changes provide more binding sites for EGCG and PD, leading to the formation of a more stable network structure through ultrasonically enhanced hydrophobic and electrostatic interactions [76], which is consistent with the circular dichroism and fluorescence spectroscopy results. The modified complex exhibited enhanced amphiphilicity, enabling it to adsorb more rapidly and firmly at the oil–water interface, thereby forming an interfacial film that prevents phase separation and droplet aggregation [72]. This accounts for the slowest particle size growth observed in the SPI-EGCG-PD(U) sample. The ζ-potential of all emulsions decreased significantly, indicating that coalescence occurred between the aqueous and oil phases during storage, leading to gradual droplet aggregation and a reduction in surface repulsive forces. The emulsion prepared by SPI-EGCG-PD(U) showed the slowest decline and maintained the highest ζ-potential, demonstrating the strongest electrostatic repulsion on the droplet surface. This is attributed to the ultrasonically promoted complex enriching more charged groups at the interface [77].

As shown in Fig. 6C, the SPI stabilized emulsion exhibited phase separation after 7 days, with white flocculent material appearing in the liquid phase. This is likely due to insufficient interfacial film strength and droplet coalescence under gravity. The SPI-EGCG stabilized emulsion showed slight aggregation, while other samples maintained uniform droplet distribution. At 15 days of storage, emulsions stabilized by SPI, SPI-EGCG, SPI-PD, and SPI-EGCG-PD all showed varying degrees of phase separation. In contrast, the emulsions prepared with ultrasonicated samples showed no phase separation, suggesting that ultrasonic treatment can effectively mitigate this phenomenon. Within the 30-day storage period, only the emulsion stabilized by SPI-EGCG-PD(U) exhibited no noticeable creaming or aggregation, indicating that the ultrasonicated ternary complex forms a denser network structure that effectively inhibits droplet aggregation [78]. Whereas, for the non-ultrasonicated samples, their loose composite structure and weak interfacial adsorption led to the disruption of the interfacial film during storage, resulting in droplet coalescence and eventual phase separation [79].

### 3.4 Correlation analysis

**Fig. 7:**
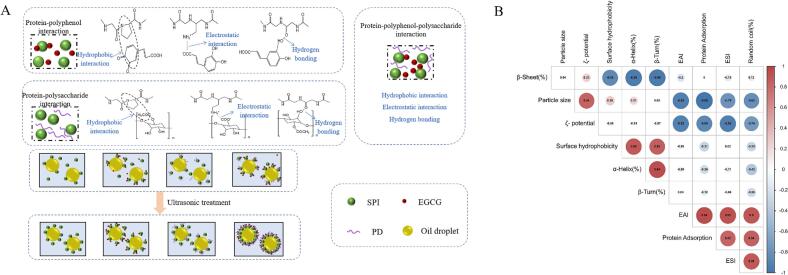
Interaction mechanisms of SPI, SPI-EGCG, SPI-PD, and SPI-EGCG-PD and their distribution at the oil–water interface in ultrasonically treated samples (A), Pearson correlations (* *P* < 0.05) (B) of samples.

Fig. 7A illustrates the mechanism diagram showing the distribution at the oil–water interface of soy protein isolate (SPI) and its binary and ternary complexes with EGCG and PD (SPI, SPI-EGCG, SPI-PD, SPI-EGCG-PD), as well as their sonicated counterparts. This diagram was derived to elucidate the non-covalent interactions and stability of these complexes within emulsions.

The effects of ultrasonic on the structure, particle size, ζ-potential, emulsifying activity index (EAI), emulsifying stability index (ESI), and adsorbed protein content at the interface of the samples, along with the relevant analysis, help summarize the mechanism of ultrasonic action. Fig. 7B illustrates the correlations between the physicochemical properties of the proteins and the stability of the emulsions, determined by the Pearson correlation coefficient (r). The value of r ranges from −1 to 1, with color coding used to indicate the strength of the correlation. The adsorption capacity of proteins at the oil–water interface is central to their emulsifying activity. Ultrasonic treatment modifies the protein structure of the prepared samples; an increase in random coil structure facilitates protein unfolding and the formation of a stable interfacial film. Consequently, EAI and ESI show significant positive correlations with interfacial adsorbed protein content and random coil structure, respectively. Ultrasonic treatment disrupts β-sheet secondary structure, induces the formation of random coil structure, reduces particle size, and increases the absolute value of ζ-potential, thereby preventing excessive protein aggregation. Therefore, these parameters exhibit significant negative correlations with β-sheet content, particle size, and ζ-potential. These findings collectively indicate that preparing protein–polyphenol-polysaccharide complexes via ultrasonic treatment has a positive effect on their emulsifying stability.

## 4 Conclusion

In this study, ultrasonic treatment was applied to SPI and its complexes (SPI-EGCG, SPI-PD, SPI-EGCG-PD). Their structures and functionalities were characterized, and subsequently, emulsions were prepared to investigate their stability, interfacial behavior, and rheological properties. The results demonstrate that the incorporation of EGCG significantly enhanced the antioxidant activity of the complexes. The addition of PD primarily improved the physical stability of the emulsions through its potent steric hindrance effect. Ultrasonic treatment, via cavitation and mechanical shear forces, significantly optimized the structural and functional properties of the protein and its complexes. The ultrasonic-induced rearrangement of protein secondary structure and exposure of hydrophobic groups enhanced the interfacial adsorption capacity and antioxidant activity of the complexes. The synergistic action within the ultrasonicated ternary complex (SPI-EGCG-PD) facilitated the formation of a denser interfacial film in the emulsion, resulting in the lowest interfacial tension (20.89 mN/m), optimal emulsion stability (89.6 min), significantly improved rheological properties, the highest oxidative stability (5.31 µmol/L) and the best storage stability. This research offers insights into the diverse modification of SPI and expands its potential applications in emulsions.

## Data availability

The data that has been used is confidential.

## CRediT authorship contribution statement

**Yuyang Huang:** Visualization, Software, Funding acquisition, Conceptualization. **Baoning Zheng:** Writing – original draft, Methodology, Formal analysis. **Bingyu Sun:** Investigation. **Ying Zhu:** Writing – review & editing. **Linlin Liu:** Visualization, Validation. **Jiyuan Liu:** Validation. **Yixin Zhang:** Investigation, Data curation. **Yang Li:** Resources, Project administration. **Xiuqing Zhu:** Writing – review & editing, Supervision.

## Declaration of competing interest

The authors declare that they have no known competing financial interests or personal relationships that could have appeared to influence the work reported in this paper.
